# Simple method to distinguish the type of fetal premature contraction using arterial Doppler time interval measurements

**DOI:** 10.1111/jog.14563

**Published:** 2020-11-08

**Authors:** Yozo Teramachi, Yasuki Maeno, Akiko Hirose, Takashi Horinouchi, Yutaka Kozuma, Toshiyuki Yoshizato, Kenji Suda

**Affiliations:** ^1^ Department of Pediatrics and Child Health Kurume University School of Medicine Kurume Japan; ^2^ Department of Obstetrics and Gynecology Kurume University School of Medicine Kurume Japan

**Keywords:** fetal arrhythmia, fetal ultrasound, premature contraction, prenatal diagnosis, sinus node

## Abstract

**Aim:**

The purpose of this study was to establish a simple method to distinguish premature ventricular contractions (PVC) from premature atrial contractions (PAC) using a fetal Doppler ultrasound arterial pulse waveform to measure time intervals between sinus node restarting.

**Methods:**

We retrospectively identified 14 fetuses with premature contraction (8 with PAC, 6 with PVC). We measured two distinct parts of time intervals using an arterial pulsed‐wave Doppler: the two consecutive waveforms just before the premature contraction (2‐V interval) and two consecutive waveforms including the premature contraction (XV interval) to measure time intervals between sinus node restarting. We then evaluated the time difference between the 2‐V and XV intervals in PVC compared to PAC.

**Results:**

For PVC, the difference between the 2‐V interval and the XV interval was significantly shorter than that for PAC. A cut‐off point of 33 ms, where a difference ≤33 ms was clearly shown to be associated with a PVC and a difference more than 33 ms signified a PAC was demonstrated.

**Conclusion:**

The 2‐V and XV interval measurements, used to measure time intervals between sinus node restarting, could easily distinguish PVC from PAC *in utero*. Therefore, this study could potentially be a feasible and effective method for obstetricians or sonographers to employ usefully.

## Introduction

Premature fetal contractions can be observed in 1–3% of all fetuses and are thought to be a relatively benign condition.^1^, ^2^ Premature atrial contractions (PAC) are 10 times more common than premature ventricular contraction. (PVC).^3^ The American Heart Association guidelines recommend that fetuses with PVC or frequent PAC that occur more than every 3–5 beats on average should have a thorough fetal echocardiogram performed.^4^ Hence, it is essential to distinguish PVC from PAC even with a low‐level examination like a fetal ultrasound.

The simultaneous recording of atrial and ventricular contractions obtained from an M‐mode, or pulsed‐wave Doppler echocardiography is widely used to distinguish between the different types of premature contractions.^5^, ^6^ M‐mode imaging obtained by placing the beam line through the atrial wall and the other side of the ventricular wall,^5^ or a simultaneous recording of Doppler waveforms in the vein and artery, can show the timing of the atrial and ventricular contractions.^6^, ^7^ However, it is often difficult to obtain sufficient imaging with low‐level examination to clearly distinguish PVC from PAC.

It is well known that the timing of a sinus atrial contraction following a PAC differs from that following a PVC.^8^ Extra electrical impulses reset the sinus node rhythm in PAC so that the atrial and ventricular contractions that follow the premature contraction appear earlier than the sinus activity is expected. Whereas with PVC, the sinus node rhythm usually is not reset and the atrial and ventricular contractions that following premature contraction usually are in time with the original sinus rhythm.

The purpose of this study was to develop a simple method to determine the type of premature contraction using this difference in the arterial flow start time after the premature contraction measured by pulsed‐wave fetal Doppler ultrasound.

## Methods

This was a retrospective observational study at a single center. We reviewed fetal ultrasound records with isolated premature contractions at Kurume University Hospital, Kurume City, Japan, between January 1, 2013 and December 31, 2017. We employed both the M‐mode and pulsed‐wave Doppler mode to diagnose of the PAC and PVC as described previously.^5^, ^6^, ^9^ In short, the pulsed Doppler recording method was simultaneously applied to the superior vena cava and the ascending aorta to distinguish a PVC from a PAC. A ProSound F75 Premier (Hitachi, Ltd) ultrasound was used to obtain the fetal ultrasound. We excluded the cases of bigeminy of premature contractions, and the cases of blocked PAC. We also excluded the cases in which the recorded artery pulse Doppler did not show any PAC or PVC.

**Figure 1:**
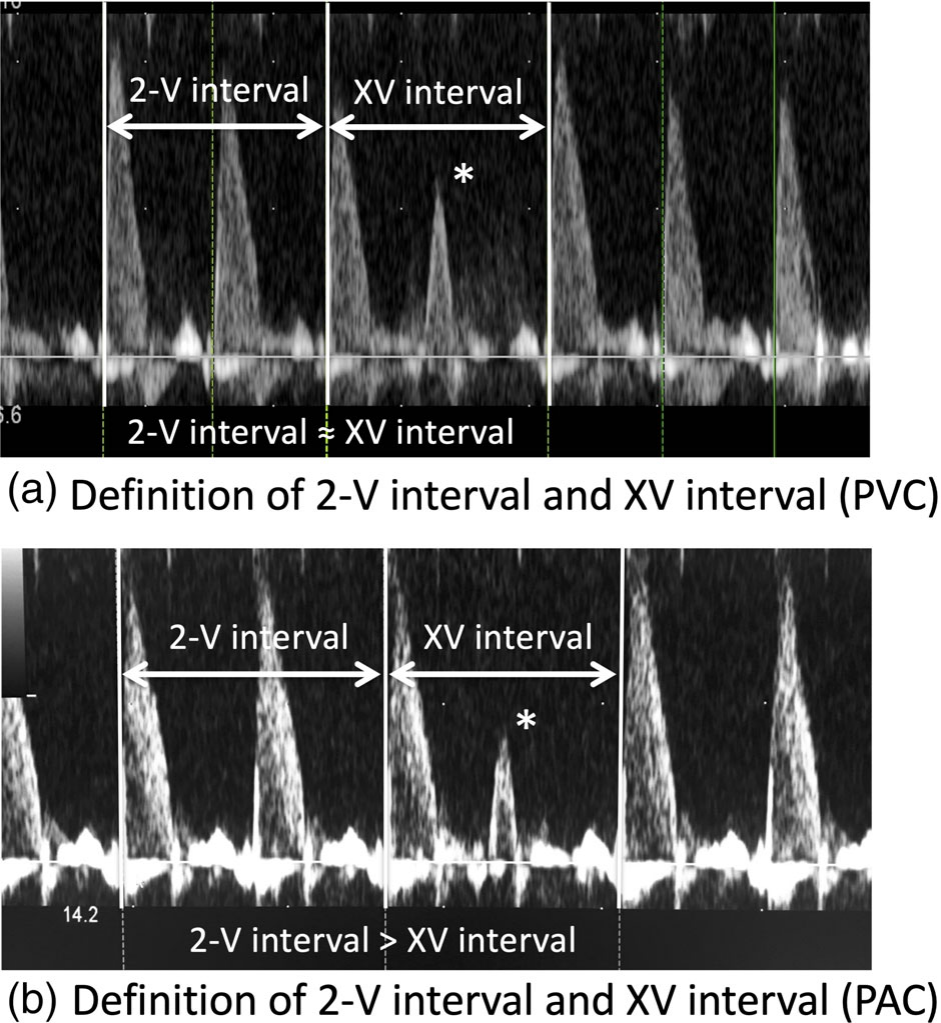
Definition of 2‐V interval and XV interval. The 2‐V interval is the time interval measurement of two consecutive regular arterial Doppler waves just before the premature contraction. The XV interval is the time interval measurement of two arterial Doppler waves, including the premature contraction. (a) A premature ventricular contraction (PVC), and (b) a premature atrial contraction (PAC). For the PVCs, the 2‐V interval was approximately equal to the XV interval. On the other hand, for the PACs, the 2V‐interval was more than the XV interval.

We used the arterial Doppler waveform obtained from the ascending aorta, the main pulmonary artery or the umbilical artery to measure the timing of contractions after the premature beat. We defined two distinct parts of time intervals measurements in the arterial Doppler spectroscopy. First, the 2‐V interval was the measurement of the two consecutive regular arterial Doppler flow waves just before the premature beat (Figure 1). Second, we measured the XV interval, the measurement of two arterial Doppler waves, which included the premature beat.

We compared the 2‐V interval to the XV interval to distinguish PVC from PAC (Figure 1). Theoretically, with PVC, the 2‐V interval and the XV interval would be almost the same since the sinus node activity would not be reset, whereas with PAC, the XV interval would be shorter than the 2‐V interval because the sinus node activity would be reset and the following contraction would appear earlier. Due to the anonymous nature of the data, informed consent was waived. This study was approved by the institutional review board of the University of Kurume (No. 17325).

### Statistical analyses

For interobserver reliability, two independent observers (Y. T. and Y. M.) analyzed the 2‐V and XV intervals of all fetuses (8 with PAC, 6 with PVC). Intraobserver reliability was calculated after a reanalysis of data from all 14 fetuses by the observer (Y. T.) 2 months after the primary analysis. For both interobserver and intraobserver reliability, the intraclass correlation coefficients were assessed and Bland–Altman analyses were performed.

We used the Wilcoxon signed‐rank test to compare the two groups (PAC and PVC). A *P*‐value <0.05 was considered to be statistically significant. We determined the cut‐off point based on sensitivity and accuracy. All analyses were performed using professional statistical software (JMP Ver. 13.0.0; SAS Institute Inc.).

## Results

**Figure 2:**
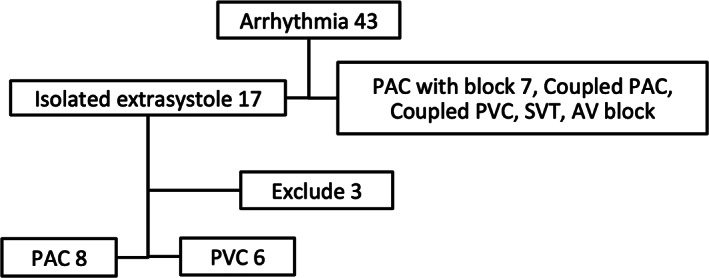
Flowchart of patient inclusion and exclusion in this study.

A total of 43 fetuses had rhythm abnormalities and were referred to our institution between April 2013 and December 2017. Of these, 17 had isolated extrasystole and we excluded 3 of these cases since no premature contraction were found on the recorded arterial pulse Doppler waveform (Figure 2). Therefore, of the 43 fetuses, 14 were included in this study, 8 with isolated PACs without a block and 6 with isolated PVC. There were no significant differences in the weeks of gestation or fetal heart rate in the fetuses with PVC and PAC (Table 1). Two fetuses with PVC had underlying cardiac diseases, one with a ventricular aneurysm and the other with a ventricular diverticulum.

**Table 1:** Background

	PAC	PVC	*P* value
Number of patients	8	6	
Gestational weeks	33.1 ± 3.9	34.8 ± 1.6	0.34
Measured site (Ao/PA/UA)	6/0/2	4/2/0	
Heart rate (bpm)	130 ± 7.0	141 ± 25	0.26
Underlying disease	0	2	

*P* values < 0.05 were statistically significant. Data are reported as mean ± SD. Ao, aortic artery; bpm, beats per minutes; *n*, number; PA, pulmonary artery; PAC, premature atrial contraction; PVC, premature ventricular contraction; SD, standard deviation; UA, umbilical artery.

**Figure 3:**
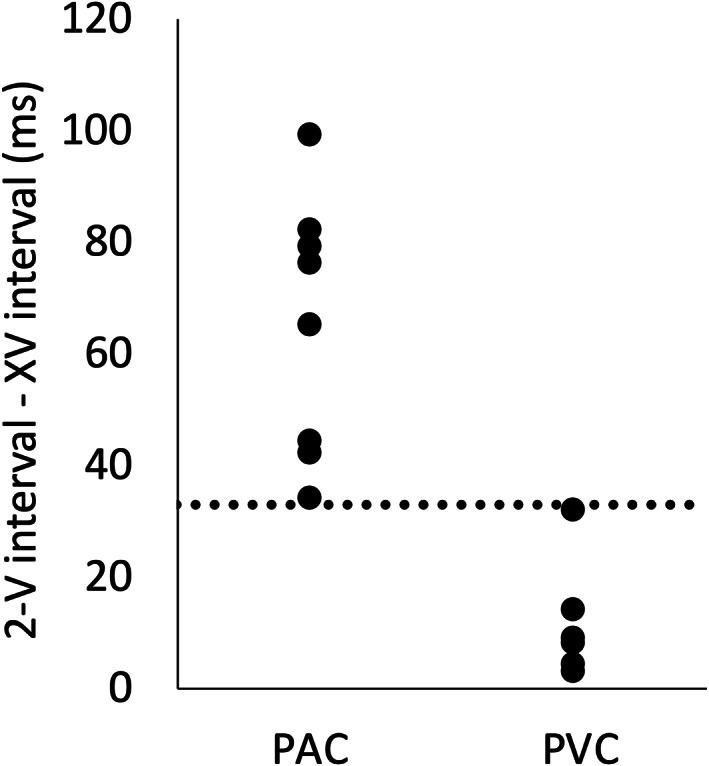
The difference between the 2‐V interval and the XV interval for premature ventricular contractions (PVC) and premature atrial contractions (PAC). The difference between the 2‐V interval and the XV interval was shorter than 33 milliseconds in all cases with PVCs and longer than 33 milliseconds in all cases with PACs.

For fetuses with PVC, the XV interval appeared to be almost equal to the 2‐V interval. The difference between the 2‐V interval and the XV interval was less than 33 ms in all fetuses with PVC. On the other hand, the difference between the 2‐V interval and the XV interval was longer than 33 ms in all fetuses with PAC (Figure 3). For fetuses with a difference that was less than 33 ms, distinguishing the fetal PVC had a sensibility and specificity of 100% (Figure 3).

**Figure 4:**
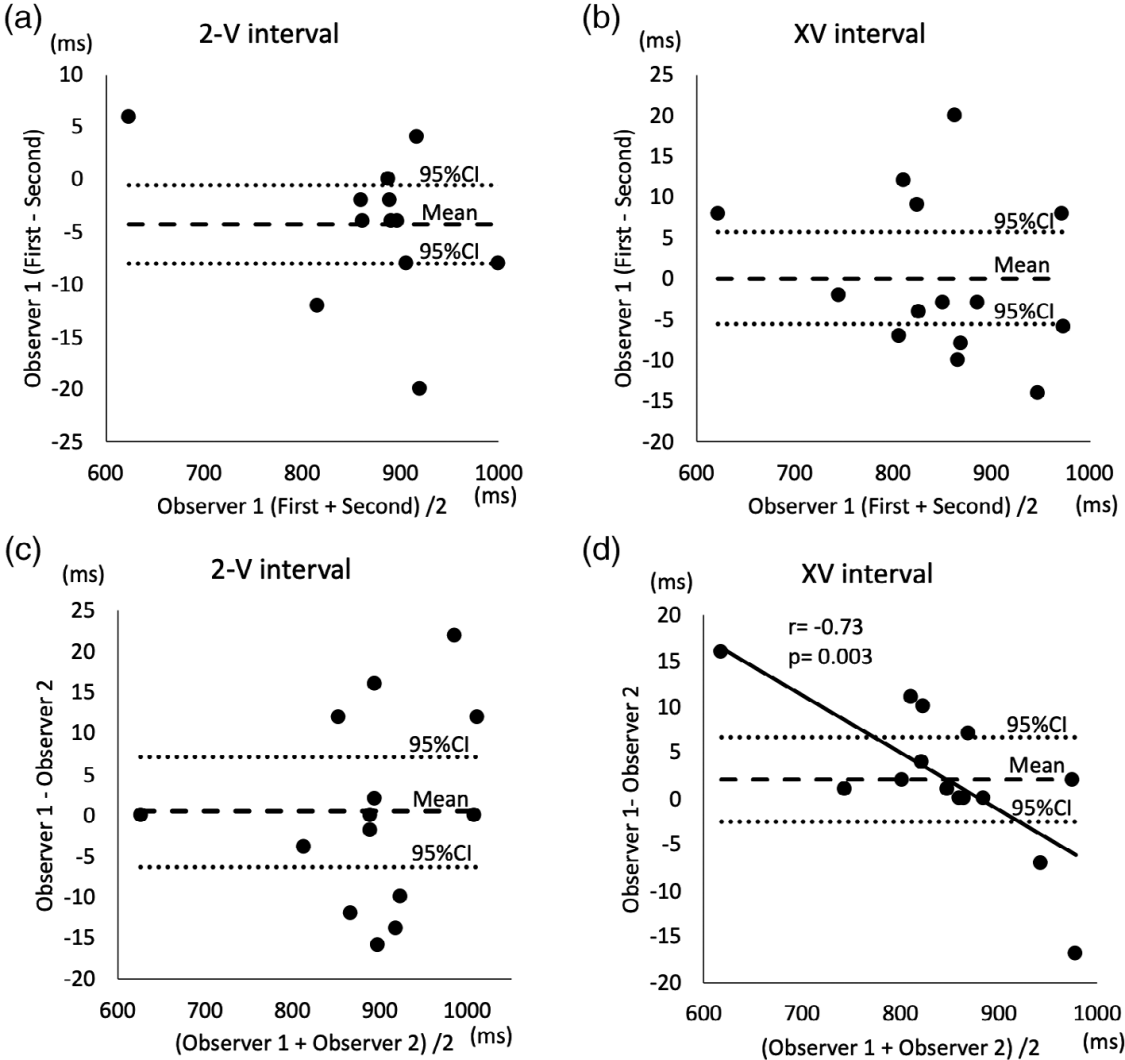
(a–d) Bland–Altman plot of interobserver and intraobserver reliability demonstrated in Tables 2 and 3. The mean of two measurement and the difference between two measurements had negative correlation (*r* = −0.73, *P* = 0.003) (d).

We did not find significant differences depending on the arterial measurement site. There were 6 ascending aorta and 2 umbilical artery cases with PAC and 4 ascending aorta and 2 pulmonary artery cases with PVC. Parameters were reproducible with sufficient accuracy at the interobserver (ICC (1)) and intraobserver (ICC (1, 2)) level (Tables 2 and 3 and Figure 4). About the XV intervals of interobserver analysis (ICC (1, 2), we found that the mean of two measurement and the difference between two measurements had negative correlation (Figure 4d).

**Table 2:** Intraobserver reliability of 2‐V interval and XV interval for all 14 fetal

		Bland–Altman‐analysis	
	(ICC (1))	(95% CI)	Correlation coefficient, *P* value
2‐V interval	0.99	−4.29 (−0.05, −8.01)	−0.33, *P* = 0.25
XV interval	0.99	0 (−5.63, 5.63)	−0.28, *P* = 0.34

Bland–Altman‐analysis; the average of difference (95% confidence interval). CI, confidence interval; ICC, intraclass correlation coefficient.

**Table 3:** Interobserver reliability of 2‐V interval and XV interval for all 14 fetal

		Bland–Altman‐analysis	
	(ICC (1, 2))	(95% CI)	Correlation coefficient, *P* value
2‐V interval	0.98	0.42 (−6.26, 7.11)	0.21, *P* = 0.48
XV interval	0.99	2.14 (−2.46, 6.74)	−0.73, *P* = 0.003

Bland–Altman‐analysis; the average of difference (95% confidence interval). CI, confidence interval; ICC, intraclass correlation coefficient.

## Discussion

We found that the time difference between the 2‐V interval and the XV interval measured using a simple arterial Doppler spectroscopy could clearly distinguish PVC from PAC. The difference between the 2‐V interval and the XV interval was less than 33 ms in all fetuses with PVC, and was more than 33 ms in all fetuses with PAC. This simple method could potentially be effective for selecting fetuses with PVC to be sent to the fetal cardiac center for further assessment.

Theoretically, the timing of an atrial contraction after a PVC differs from that after a PAC. Unlike PAC, the sinus node activity is not reset with a PVC and the atrial and ventricular contractions after the premature contraction, therefor, remains in the original sinus rhythm. Our study demonstrated that this theoretical time difference between PVC and PAC could be measured by arterial fetal Doppler ultrasound and has the potential to become a simple method to differentiate the type of premature contraction.

The crucial advantage of our presented method is that it is a simple technique that does not require live images of atrial movement. All of the conventional methods such as the M‐mode method, the pulsed‐wave Doppler method (obtained in the superior vena cava, ascending aorta, hepatic vein^7^ or pulmonary artery or vein), and the tissue Doppler method require the simultaneous recording of atrial contractions and ventricular contractions to distinguish PVC from PAC.^10^, ^11^ However, it is often difficult to obtain sufficient imaging, especially when the fetal position is not unsuitable and/or premature contractions do not appear frequently. Additionally, not all sonographers at screening centers are familiar with these techniques. PVC and PAC should be distinguished at low‐level centers, since the American Heart Association guidelines recommended that PVC be referred to the fetal echocardiogram center even when the premature contractions are not frequent.^4^ In fact, neither of the two patients who were diagnosed with ventricular diverticulum and aneurysm by fetal echocardiography in our center were detected at the time of referral. Hence, our simple method of assessing ventricular contractions by Doppler arterial waveform may become a feasible technique for low‐level centers.

Additionally, this method has other advantages. For instance, it does not require a particular fetal position since the Doppler flow wave in any artery can be selected for measurement. We measured the Doppler spectroscopy in the ascending aorta, the pulmonary artery and the umbilical artery. This method clearly distinguishes the PVC from the PAC despite the artery measurement site. Furthermore, this simple technique with low interobserver reliability does not require exceptional sonographer skills. Hence, this method can easily and widely be performed at any low‐level center without advanced training.

However, several limitations should be recognized in the present study. First, this was a retrospective observational study performed at a single facility. The limited number of cases did not allow for an analysis of data obtained at different gestational weeks or with various fetal heart rates. Another limitation is that the arterial site we selected (ascending aorta, pulmonary or the umbilical artery) depended on the available recorded Doppler waveforms, which included the premature contractions. The third limitation was the interreliability and intrareliability. The difference between observer suggested that measurements may depend on fetal heart rate. Further prospective studies with a larger number of fetuses are required to reconfirm the definitive cut‐off level in various arterial sites.

This study showed that 2‐V interval and the XV interval measurements from arterial Doppler waveforms using fetal ultrasound can easily be used to distinguish PVC form PAC *in utero*. This simple technique may be a feasible method to select the fetuses with PVC requiring further assessment at the fetal cardiac center.

## Disclosure

None declared.
